# Pylorus Preserving Pancreaticoduodenectomy After Prior Esophagogastrectomy

**DOI:** 10.1089/pancan.2020.0014

**Published:** 2020-12-10

**Authors:** Rachel Appelbaum, Daniel C. Kuehler, Jeffrey Brodsky

**Affiliations:** ^1^ Department of Surgery and Lehigh Valley Health Network, Allentown, Pennsylvania, USA.; ^2^ Department of Surgical Oncology, Lehigh Valley Health Network, Allentown, Pennsylvania, USA.

**Keywords:** adenocarcinoma, carcinoma, pancreatic cancer, surgery

## Abstract

**Background:** As treatment of esophageal carcinomas continues to improve, we have seen an increasing population of long-term survivors giving rise to the observation of additional primary malignancies not previously seen. Esophagogastrectomy for previously treated esophageal carcinoma presents unique anatomic changes providing further technical difficulties for surgical management of new primary malignancies.

**Presentation:** A 65-year-old male with a history of esophagogastrectomy for esophageal adenocarcinoma presents with a pancreatic head mass consistent with pancreatic adenocarcinoma. Our case report describes a pylorus sparing pancreaticoduodenectomy with preservation of the right gastric and right gastroepiploic vessels in order to preserve blood supply to the gastric conduit.

**Conclusion:** Here we demonstrate that in select cases where location of the pancreatic head tumor is favorable, pancreaticoduodenectomy can be performed in the context of prior esophagogastrectomy with preservation of the native blood supply to the gastric conduit. Pancreaticoduodenectomy may have yet been possible if the tumor involved the gastroduodenal artery via vascular reconstruction to the right gastroepiploic artery or sacrifice of the gastric conduit with reconstruction using small or large intestine.

## Background

Development of a periampullary malignancy after prior esophagogastrectomy is a rare event. However, progress with early detection and multidisciplinary treatment has led to an increasing population of long-term esophageal cancer survivors.^1,2^ Pancreaticoduodenectomy after esophagogastrectomy presents unique technical challenges related to preservation of blood supply to the gastric conduit. Our case report describes a pylorus sparing pancreaticoduodenectomy with preservation of the right gastric and right gastroepiploic vessels.

## Case Description

A 65-year-old male was referred for a malignant head of pancreas mass. He was diagnosed with adenocarcinoma of the middle esophagus in 2018. After neoadjuvant chemoradiation, he underwent esophagogastrectomy through an abdominal and right thoracic approach. There was a complete pathological response. A routine surveillance computed tomography scan 1 year after esophageal surgery revealed a 2 cm mass in the head of the pancreas without biliary or pancreatic ductal dilatation. The gastroduodenal artery was not involved. This was also visualized on magnetic resonance imaging.

A positron emission tomography scan showed mild uptake of the pancreatic mass with maximum standardized uptake value of 3.5, and no metastatic disease. Endoscopic ultrasound confirmed a 2 cm head of pancreas mass. Biopsy provided cytology positive for adenocarcinoma, presumed to be a new primary tumor as opposed to metastatic disease, clinical stage T2N0M0.

The treatment plan was to proceed to pylorus sparing pancreaticoduodenectomy, with attempt to preserve the native blood supply to the gastric conduit. Vascular surgery was on standby in the event that a graft would be required to the right gastroepiploic artery.

**FIG. 1.**
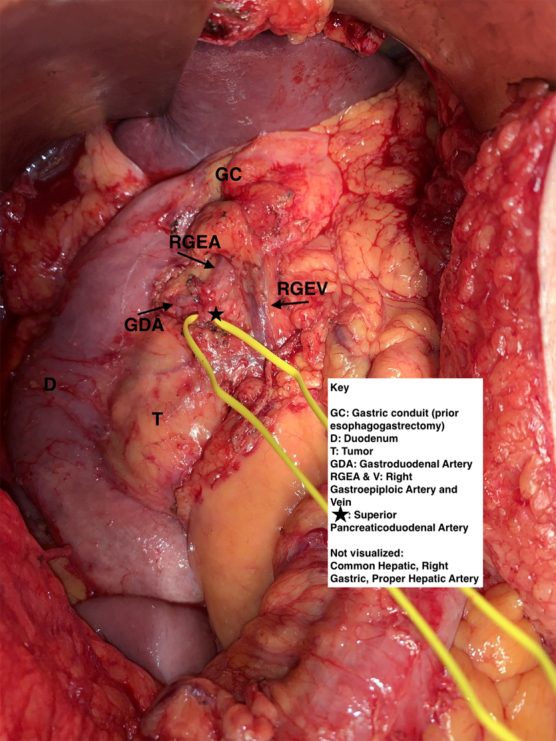
Intraoperative anatomy with superior pancreaticoduodenal artery isolated, preserving gastroduodenal artery.

**FIG. 2.**
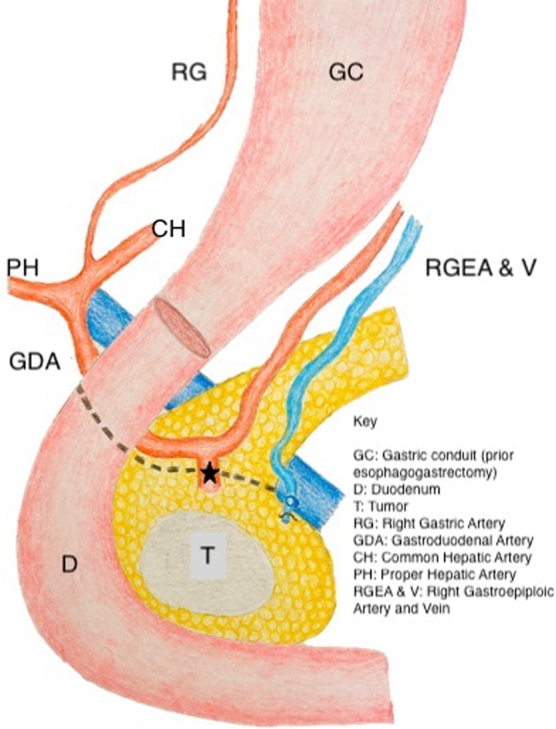
Artistic rendition of the outlined anatomy.

At operation, the gastroduodenal artery was exposed and traced to its bifurcation into the right gastroepiploic and superior pancreaticoduodenal vessels. The superior pancreaticoduodenal artery was looped and staple transected, Figure 1. The middle colic vein branch of the gastrocolic vein trunk was divided, whereas the right gastroepiploic vein was preserved, Figure 1. The transection line of the pancreas is demonstrated diagrammatically in Figure 2.

The pancreatic duct was 1 mm and the gland was soft with poor suture-holding capability, precluding the usual pancreatic anastomotic technique used at our institution. A short segment of 5 Fr pediatric feeding tube was inserted into pancreas and jejunum and fixed in place with absorbable suture. Four tacking sutures were placed between the jejunal serosa and pancreatic capsule. Operative time was 310 min.

The postoperative course was notable for a pancreatic fistula from one of the operatively placed drains. This was treated with antibiotics and octreotide. Discharge was on postoperative day 15 and the drain was removed in the office on postoperative day 23. Pathology demonstrated a T2 (2.9 cm) N1 (1/23 lymph nodes) moderately differentiated pancreatic adenocarcinoma. All margins were negative, including the radial, anterior pancreatic, and retroperitoneal uncinate margins. Adjuvant therapy was initiated with fluorouracil, irinotecan, oxaliplatin, and leucovorin, which was poorly tolerated and discontinued. The patient is currently 8 months out from surgery and clinically stable.

## Conclusion

In select cases where location of the pancreatic head tumor is favorable, pancreaticoduodenectomy can be performed in the context of prior esophagogastrectomy with preservation of the native blood supply to the gastric conduit.

## Discussion

Earlier detection and improved multimodal treatment strategies have improved survival for esophageal cancer patients. With more long-term survivors, increased frequency of second malignancies of the upper gastrointestinal tract should be expected.^1^ Second malignancies of the periampullary region pose significant surgical challenges, related to preservation of blood supply to the gastric conduit.^1,2^

For select tumors, pancreaticoduodenectomy can be performed with preservation of the right gastric and right gastroepiploic vessels.^1–4^ If the tumor involves the gastroduodenal artery, options include vascular reconstruction to the right gastroepiploic artery or sacrifice of the gastric conduit with reconstruction using small or large intestine.^2,4^ Precise anatomical study of the primary tumor and vascular anatomy is critical to successful surgery.
